# Beyond the abdomen: an interpretable machine learning model for predicting postoperative ileus in non-abdominal surgery

**DOI:** 10.3389/fphys.2026.1814394

**Published:** 2026-06-12

**Authors:** Lingjun Chen, Zihang Ma, Xiaoting Zhang, Xuezheng Lin, Lin Wang

**Affiliations:** 1 Department of Anesthesia Surgery, Taizhou Central Hospital (Taizhou University Hospital), Taizhou, China; 2 The First Clinical Medical School of Ningxia Medical University, Yinchuan, China

**Keywords:** brain-gut axis, machine learning, non-abdominal surgery, postoperative ileus, selective serotonin reuptake inhibitors

## Abstract

**Purpose:**

Postoperative ileus (POI) following non-abdominal surgery is an underestimated complication. This study aimed to develop and validate a machine learning model to predict POI risk, specifically integrating brain-gut axis variables, including depression history and chronic selective serotonin reuptake inhibitor (SSRI) use.

**Methods:**

A multicenter retrospective study included 2000 patients undergoing non-abdominal surgery. The cohort was divided into training (n=1050), internal testing (n=450), and external validation (n=500) cohorts. A dual-algorithm feature selection strategy combining LASSO and Boruta was used to identify robust predictors. Eight machine learning algorithms were developed and compared. Model performance was evaluated using the Area Under the Curve (AUC), calibration plots, and Decision Curve Analysis.

**Results:**

Seven independent predictors were identified: chronic SSRI use, history of depression, intraoperative opioids, duration of surgery, neutrophil-to-lymphocyte ratio, serum albumin, and fluid balance. The Random Forest model demonstrated superior discrimination, achieving an AUC of 0.942 in the training cohort, 0.917 in the internal testing cohort, and 0.895 in the external validation cohort. It significantly outperformed standard logistic regression (p<0.05) and displayed excellent calibration. Decision curve analysis indicated a high net clinical benefit, while SHAP analysis visually confirmed the substantial contribution of brain-gut axis factors to delayed bowel recovery.

**Conclusion:**

The Random Forest model provides a robust and generalizable tool for predicting POI in non-abdominal surgery patients. By highlighting the critical influence of the brain-gut axis, this study offers new insights for risk stratification and personalized perioperative management.

## Introduction

Postoperative ileus is one of the most common and frequent complications following surgery (Xue et al., 2021). It is characterized by a transient impairment of bowel motility that prevents the effective transit of intestinal contents (Humphry et al., 2023). This condition leads to significant perioperative morbidity, including prolonged hospitalization, increased healthcare costs, and a higher risk of pulmonary complications and surgical site infections (Song et al., 2022). While postoperative ileus is extensively studied in the context of abdominal surgery where direct bowel manipulation occurs, its occurrence in non-abdominal surgery is often underestimated. However, the systemic stress response, anesthesia, and pharmacological interventions associated with non-abdominal procedures can still precipitate substantial gastrointestinal dysfunction, necessitating reliable risk stratification strategies for this specific patient population (Deo et al., 2022).

The pathophysiology of postoperative ileus is multifactorial, involving neurogenic, inflammatory, and pharmacological mechanisms (Jin et al., 2023). Recently, the bidirectional communication between the central nervous system and the enteric nervous system, known as the brain-gut axis, has garnered increasing attention (Meade et al., 2022). Serotonin, a key neurotransmitter regulating mood, also plays a critical role in modulating gastrointestinal motility (Chang et al., 2014). Consequently, preoperative psychological states such as depression and the chronic use of psychotropic medications like selective serotonin reuptake inhibitors (SSRI) may profoundly influence postoperative bowel recovery. Despite this biological plausibility, few predictive models have explicitly integrated these brain-gut axis variables into the risk assessment of postoperative ileus.

Traditional predictive models typically rely on conventional logistic regression analysis. While useful, these linear models often struggle to capture complex, non-linear interactions among high-dimensional clinical variables. In contrast, machine learning algorithms offer a superior alternative by efficiently processing large datasets and identifying subtle patterns that escape standard statistical methods. Furthermore, advanced feature selection techniques and model interpretation tools, such as SHapley Additive exPlanations, help bridge the gap between complex algorithmic predictions and clinical comprehensibility.

Therefore, the primary aim of this study was to develop and validate a robust machine learning model to predict the risk of postoperative ileus in patients undergoing non-abdominal surgery. We specifically sought to investigate the predictive value of brain-gut axis indicators, including a history of depression and chronic SSRI use. By employing a rigorous feature selection strategy based on the intersection of LASSO and Boruta algorithms, and by validating the model in an independent external cohort, this study intends to provide clinicians with an accurate and interpretable tool to optimize perioperative management and patient outcomes.

## Materials and methods

### Study design and participants

This retrospective cohort study was conducted at two independent medical centers between January 2018 and December 2025. The study protocol was approved by the Institutional Review Board, and the requirement for informed consent was waived due to the retrospective nature of the analysis (Approval No. 2026L-03-60). We recruited patients who underwent non-abdominal surgeries under general anesthesia. Taizhou Central Hospital (Taizhou University Hospital) served as the primary center for model development, where patients were randomly divided into a training cohort and an internal testing cohort using a 70:30 ratio. The Fourth Affiliated Hospital of Soochow University served as an independent center to provide an external validation cohort, ensuring the generalizability of the developed models.

We applied strict inclusion and exclusion criteria to ensure data quality. Patients aged 18 years or older were included in the study. We excluded patients who had preoperative bowel obstruction, underwent emergency surgery, or required a second operation during the same hospitalization. Additionally, patients with significant missing data exceeding 20% of the key variables were excluded from the final analysis.

### Outcome definition and data collection

The primary outcome of this study was the incidence of postoperative ileus. The diagnosis of postoperative ileus was determined through a comprehensive clinical assessment. Specifically, it was defined as the absence of flatus for more than four days after surgery, combined with clinical symptoms such as abdominal distension, nausea, and vomiting, as well as patient subjective complaints indicating bowel dysfunction (Chapman et al., 2018). This specific >4 days threshold was chosen to define clinically significant “prolonged” postoperative ileus (PPOI). According to previous global consensus and literature (Vather et al., 2013), mild bowel delays resolving within 3–4 days are often considered a transient, physiological response to anesthesia and perioperative opioids. By defining POI as >4 days, our model specifically targets patients who are at risk of prolonged recovery, which is strictly associated with increased morbidity, prolonged hospitalization, and the need for medical intervention, thereby maximizing the clinical utility of the predictive model. To minimize misclassification bias caused by varying practice patterns across centers, data extraction was highly standardized. Two independent researchers at each center systematically reviewed electronic medical records, including nursing flowsheets, physician progress notes, and medication records, using uniform diagnostic criteria. Any ambiguous clinical documentation or discrepancies between reviewers were resolved through consensus with a senior clinician.

Data were extracted from the electronic medical record systems of the participating hospitals. We collected a total of 38 potential predictor variables for each patient as detailed in **Table 1**. These variables included demographic characteristics, preoperative comorbidities, intraoperative parameters, and postoperative medications. Given the focus of our study on the brain-gut axis, we specifically extracted data regarding the history of depression and the chronic use of selective serotonin reuptake inhibitors to evaluate their predictive value.

**Table 1:** Variable assignments.

Variables	Risk factors	Assignment
X1	Age	Continuous variable
X2	Gender	Male=1, Female=0
X3	BMI	Continuous variable
X4	ASA Classification	Continuous variable (1–5)
X5	Smoking history	Yes=1, No=0
X6	Preoperative opioid use	Yes=1, No=0
X7	History of anxiety or depression	Yes=1, No=0
X8	Chronic SSRI use	Yes=1, No=0
X9	Preoperative sleep quality score (PSQI)	Continuous variable
X10	Preoperative cognitive score (MMSE)	Continuous variable
X11	History of motion sickness	Yes=1, No=0
X12	Intraoperative BIS value (Mean)	Continuous variable
X13	Diabetes Mellitus	Yes=1, No=0
X14	Hypertension or CVD	Yes=1, No=0
X15	COPD	Yes=1, No=0
X16	History of neurological disease	Yes=1, No=0
X17	Serum Albumin	Continuous variable
X18	Neutrophil-to-lymphocyte ratio (NLR)	Continuous variable
X19	C-reactive protein (CRP)	Continuous variable
X20	Hemoglobin	Continuous variable
X21	Preoperative potassium	Continuous variable
X22	eGFR	Continuous variable
X23	Fasting blood glucose	Continuous variable
X24	Duration of surgery	Continuous variable
X25	Duration of anesthesia	Continuous variable
X26	Spine surgery	Yes=1, No=0
X27	Intraoperative opioids (MME)	Continuous variable
X28	Anesthesia maintenance type	Inhalational=1, TIVA = 0
X29	Duration of hypotension (MAP < 65mmHg)	Continuous variable
X30	Vasopressor use	Yes=1, No=0
X31	NMBA reversal agent	Sugammadex=1, Neostigmine=0
X32	Fluid balance	Continuous variable
X33	Estimated blood loss	Continuous variable
X34	Postoperative PCIA use	Yes=1, No=0
X35	Pain score at POD1 (VAS)	Continuous variable
X36	Time to first mobilization	Continuous variable
X37	Postoperative potassium	Continuous variable
X38	Perioperative blood transfusion	Yes=1, No=0
Y	Postoperative Ileus	Yes=1, No=0

BMI, body mass index; ASA, American Society of Anesthesiologists; SSRI, selective serotonin reuptake inhibitors; PSQI, Pittsburgh sleep quality index; MMSE, mini-mental state examination; BIS, bispectral index; CVD, cardiovascular disease; COPD, chronic obstructive pulmonary disease; NLR, neutrophil-to-lymphocyte ratio; CRP, C-reactive protein; eGFR, estimated glomerular filtration rate; MME, morphine milligram equivalents; TIVA, total intravenous anesthesia; MAP, mean arterial pressure; NMBA, neuromuscular blocking agent; PCIA, patient-controlled intravenous analgesia; POD1, postoperative day 1; VAS, visual analog scale.

### Data preprocessing and feature selection

Prior to analysis, the raw data underwent preprocessing to handle missing values and standardize the format. After excluding patients with significant missingness (>20%), the missing rates for all remaining individual variables were extremely low (all < 5%). The detailed missing rates for each variable are reported in **Supplementary Table 2**. An analysis of the missing data patterns suggested that the mechanism of missingness was predominantly Missing Completely at Random (MCAR) or Missing at Random (MAR), primarily attributable to random administrative omissions in the electronic medical records. For continuous variables with missing data, we employed mean imputation, while missing categorical values were filled using the mode imputation method. This single-imputation approach was chosen for its computational efficiency and to streamline the preprocessing pipeline across multiple machine learning algorithms. Its use was justified by the relatively low overall proportion of missing data, as patients with extensive missingness (>20%) had already been excluded. Continuous features were subsequently standardized using Z-score normalization to ensure that all variables contributed equally to the model.

We employed a rigorous two-step feature selection strategy to identify the most relevant predictors and eliminate redundancy. First, we utilized the Least Absolute Shrinkage and Selection Operator regression algorithm, which applies a penalty to shrink coefficients and select variables. Second, we applied the Boruta algorithm, a random forest-based method that compares original features against randomized shadow features to determine statistical significance. The final set of predictors was determined by taking the intersection of the variables selected by both the LASSO and Boruta algorithms. To ensure the independence of the selected features, we performed both Spearman correlation analysis and Variance Inflation Factor assessment. This dual approach allowed us to rigorously rule out multicollinearity among the predictors.

### Machine learning model development

We developed and compared eight distinct machine learning algorithms to predict the risk of postoperative ileus. These algorithms included Random Forest, XGBoost, LightGBM, Logistic Regression, Naive Bayes, Decision Tree, Support Vector Machine, and K-Nearest Neighbors. To optimize the performance of each model, we conducted hyperparameter tuning using grid search combined with 10-fold cross-validation on the training dataset. This process involved systematically testing various combinations of parameters to identify the configuration that yielded the best predictive accuracy while preventing overfitting.

### Model evaluation

The performance of the models was evaluated comprehensively in the training, internal testing, and external validation cohorts. We primarily used the Receiver Operating Characteristic curve and the Area Under the Curve to assess the discrimination ability of the models. We also calculated other key performance metrics including sensitivity, specificity, accuracy, and the F1-score. In addition to discrimination metrics and visual calibration curves, the Brier score was calculated for all evaluated models as a quantitative measure of calibration. The Brier score evaluates the mean squared difference between the predicted probabilities and the actual observed outcomes, with a lower score (closer to 0) indicating superior calibration.

Statistical comparisons between the Area Under the Curve values of different models were performed using the DeLong test, with a two-sided P value of less than 0.05 considered statistically significant. Furthermore, we assessed the calibration of the models using calibration curves to examine the agreement between predicted probabilities and observed outcomes. The clinical utility of the models was evaluated using Decision Curve Analysis, which quantifies the net benefit of using the model across a range of threshold probabilities.

### Model interpretability

To address the “black box” nature of machine learning models and enhance clinical transparency, we utilized SHapley Additive exPlanations. This method assigns an importance value to each feature for a particular prediction. We generated global importance plots to rank the overall contribution of each variable and summary plots to visualize the direction and magnitude of the feature effects. Additionally, waterfall plots were created to demonstrate how specific risk factors contributed to the predicted probability for individual patients, facilitating personalized clinical interpretation.

### Statistical analyses

All statistical analyses and data visualizations were performed using.

R (version 4.4.2). Continuous variables were assessed for normality via the Shapiro-Wilk test. Normally distributed data are presented as mean ± standard deviation, with group comparisons conducted using Student’s *t*-tests. Non-normally distributed variables are expressed as median and interquartile range [M (Q1, Q3)] and analyzed via the Mann-Whitney *U* test. Categorical variables are reported as frequencies (percentages) and evaluated using Chi-square tests or Fisher’s exact tests (for cell counts <5). Statistical significance was defined as a two-tailed *p*-value < 0.05.

## Result

### Baseline characteristics and variable definitions

A total of 2000 patients undergoing non-abdominal surgery were included in this study and randomly allocated into three distinct cohorts. These consisted of a training set with 1050 patients, an internal testing set with 450 patients, and an external validation set with 500 patients. **Table 2** details the baseline demographic and clinical characteristics. The average age of patients in the training cohort was 59.4 ± 11.5 years, which was comparable to the testing and validation cohorts with P values exceeding 0.05. The overall incidence of postoperative ileus was 15.4% in the training set, 16.9% in the testing set, and 14.2% in the validation set, showing no statistically significant difference with a P value of 0.485. Similarly, key brain-gut axis variables such as the history of depression and chronic SSRI use were balanced across groups, with prevalence rates of approximately 13.5% and 6.5% in the training cohort respectively. These results confirm that the randomization process effectively balanced the baseline features.

**Table 2:** Baseline characteristics of patients in the training, testing, and validation cohorts.

Characteristic	Training cohort (n = 1050)	Testing cohort (n = 450)	Validation cohort (n = 500)	P value
Demographics				
Age (years), Mean ± SD	59.4 ± 11.5	60.8 ± 10.9	58.7 ± 12.1	0.076
Gender (Male), n (%)	512 (48.8%)	234 (52.0%)	229 (45.8%)	0.143
BMI (kg/m²), Mean ± SD	24.5 ± 3.2	24.9 ± 3.5	24.2 ± 3.1	0.089
ASA classification, n (%)				0.215
I - II	785 (74.8%)	318 (70.7%)	382 (76.4%)	
III - IV	265 (25.2%)	132 (29.3%)	118 (23.6%)	
Smoking history (Yes), n (%)	258 (24.6%)	129 (28.7%)	110 (22.0%)	0.094
Preoperative opioid use (Yes), n (%)	86 (8.2%)	45 (10.0%)	34 (6.8%)	0.187
Gut-brain axis factors				
History of depression (Yes), n (%)	142 (13.5%)	74 (16.4%)	61 (12.2%)	0.156
Chronic SSRI use (Yes), n (%)	68 (6.5%)	36 (8.0%)	28 (5.6%)	0.342
Sleep quality score (PSQI), Mean ± SD	7.4 ± 3.5	7.8 ± 3.8	7.2 ± 3.3	0.128
Cognitive score (MMSE), Mean ± SD	27.5 ± 2.1	27.2 ± 2.4	27.6 ± 1.9	0.203
History of motion sickness (Yes), n (%)	195 (18.6%)	75 (16.7%)	108 (21.6%)	0.165
Intraoperative BIS (Mean), Mean ± SD	48.5 ± 5.6	47.9 ± 6.1	49.1 ± 5.2	0.068
Comorbidities				
Diabetes Mellitus (Yes), n (%)	176 (16.8%)	88 (19.6%)	75 (15.0%)	0.189
Hypertension/CVD (Yes), n (%)	438 (41.7%)	199 (44.2%)	195 (39.0%)	0.274
COPD (Yes), n (%)	65 (6.2%)	36 (8.0%)	26 (5.2%)	0.211
Neurological disease history (Yes), n (%)	48 (4.6%)	28 (6.2%)	19 (3.8%)	0.235
Preoperative labs				
Serum Albumin (g/L), Mean ± SD	39.5 ± 4.2	39.1 ± 4.5	39.8 ± 4.0	0.154
NLR, Mean ± SD	2.45 ± 1.12	2.58 ± 1.25	2.39 ± 1.05	0.092
CRP (mg/L), Median (IQR)	3.5 (1.8-6.2)	3.9 (2.0-6.8)	3.2 (1.6-5.9)	0.118
Hemoglobin (g/L), Mean ± SD	132.5 ± 15.6	129.8 ± 16.4	133.4 ± 14.8	0.072
Preoperative potassium (mmol/L), Mean ± SD	4.05 ± 0.35	4.02 ± 0.38	4.08 ± 0.32	0.165
eGFR (mL/min), Mean ± SD	88.5 ± 18.2	86.4 ± 19.5	89.2 ± 17.6	0.246
Fasting blood glucose (mmol/L), Mean ± SD	5.8 ± 1.5	6.1 ± 1.8	5.7 ± 1.4	0.082
Intraoperative variables				
Duration of surgery (min), Mean ± SD	165.4 ± 45.2	172.1 ± 48.6	160.5 ± 42.8	0.058
Duration of anesthesia (min), Mean ± SD	205.4 ± 55.2	214.5 ± 58.6	199.2 ± 50.8	0.064
Spine surgery, (Yes),n (%)	388 (37.0%)	185 (41.1%)	172 (34.4%)	0.096
Intraoperative opioids (MME, mg), Mean ± SD	45.8 ± 12.5	47.2 ± 13.8	44.9 ± 11.6	0.137
Anesthesia maintenance, n (%)				0.312
Inhalational	651 (62.0%)	268 (59.6%)	322 (64.4%)	
TIVA	399 (38.0%)	182 (40.4%)	178 (35.6%)	
Duration of hypotension (min), Median (IQR)	10 (0-25)	12 (0-28)	8 (0-22)	0.145
Vasopressor use (Yes), n (%)	345 (32.9%)	162 (36.0%)	151 (30.2%)	0.178
NMBA Reversal (Sugammadex), n (%)	498 (47.4%)	230 (51.1%)	228 (45.6%)	0.254
Fluid balance (mL), Mean ± SD	1250 ± 450	1310 ± 480	1215 ± 420	0.063
Estimated blood loss (mL), Median (IQR)	200 (100-350)	220 (120-380)	190 (100-320)	0.088
Postoperative outcomes				
PCIA use (Yes), n (%)	815 (77.6%)	338 (75.1%)	402 (80.4%)	0.162
Pain score at POD1 (VAS), Mean ± SD	3.4 ± 1.2	3.6 ± 1.4	3.3 ± 1.1	0.065
Time to first mobilization (h), Mean ± SD	22.5 ± 6.8	23.4 ± 7.5	21.9 ± 6.2	0.074
Postoperative potassium (mmol/L), Mean ± SD	3.85 ± 0.42	3.81 ± 0.45	3.88 ± 0.40	0.193
Perioperative transfusion (Yes), n (%)	115 (11.0%)	62 (13.8%)	48 (9.6%)	0.104
Outcome: Postoperative Ileus, (Yes), n (%)	162 (15.4%)	76 (16.9%)	71 (14.2%)	0.485

Data are presented as n (%) for categorical variables and as mean ± SD or median (IQR) for continuous variables, as appropriate. The symbol (%) denotes the percentage of patients within the corresponding cohort.

BMI, body mass index; ASA, American Society of Anesthesiologists; SSRI, selective serotonin reuptake inhibitors; PSQI, Pittsburgh sleep quality index; MMSE, mini-mental state examination; BIS, bispectral index; CVD, cardiovascular disease; COPD, chronic obstructive pulmonary disease; NLR, neutrophil-to-lymphocyte ratio; CRP, C-reactive protein; eGFR, estimated glomerular filtration rate; MME, morphine milligram equivalents; TIVA, total intravenous anesthesia; MAP, mean arterial pressure; NMBA, neuromuscular blocking agent; PCIA, patient-controlled intravenous analgesia; POD1, postoperative day 1; VAS, visual analog scale.

We collected 38 potential predictors for each patient to construct the model. **Table 1** provides a comprehensive list of these variables along with their assignment criteria. The candidate features encompassed diverse categories including demographic factors like age and gender, preoperative comorbidities such as diabetes and hypertension, and specific intraoperative metrics including the duration of surgery and intraoperative opioid dosage. Categorical variables such as gender and smoking history were encoded as binary values, while continuous variables like BMI and laboratory indicators retained their original numerical measurements.

### Feature selection via intersection of algorithms and collinearity check

**Figure 1:**
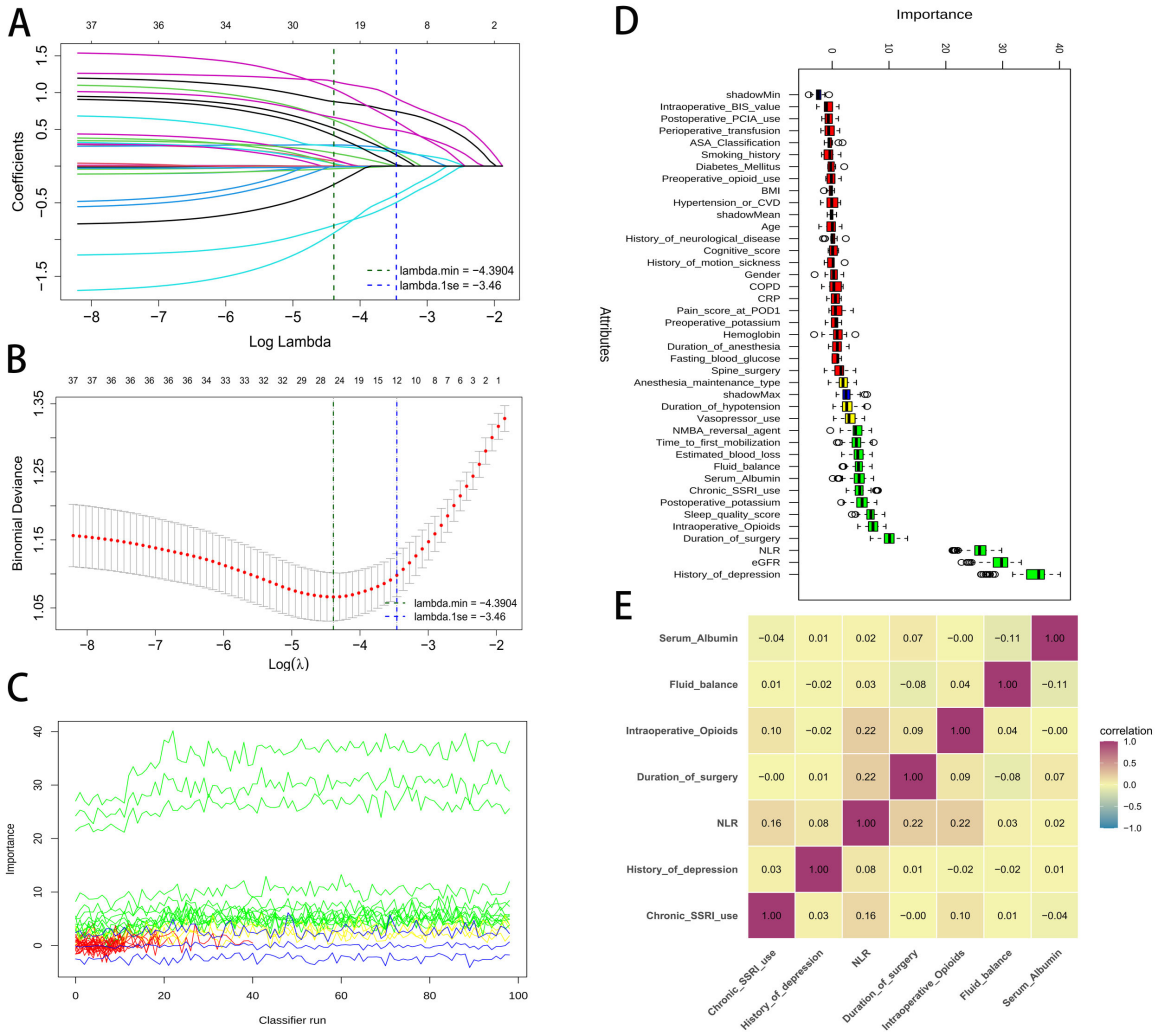
Feature selection and correlation analysis. **(A)** LASSO coefficient profiles of candidate variables. **(B)** Selection of the optimal lambda (λ*λ*) in the LASSO model using 10-fold cross-validation. **(C)** Dynamic feature importance evolution during the Boruta algorithm process. **(D)** Final feature importance ranking by Boruta; green box plots indicate confirmed predictors. **(E)** Spearman correlation heat map of the seven selected variables.

To isolate the most robust predictors from the 38 candidate variables, we implemented a dual-algorithm selection strategy. The Least Absolute Shrinkage and Selection Operator regression was first applied to minimize overfitting. As illustrated in **Figure 1A**, the coefficient profiles of the variables converged as the penalty parameter increased. The optimal lambda value was determined via 10-fold cross-validation as shown in **Figure 1B**. Concurrently, the Boruta algorithm was utilized to evaluate feature importance relative to randomized shadow attributes. **Figure 1C** depicts the selection process over multiple iterations, and **Figure 1D** highlights the final confirmed attributes that performed significantly better than the shadow maxima.

We determined the final model predictors by identifying the intersection of variables selected by both LASSO and Boruta. This rigorous process yielded seven core variables: history of depression, chronic SSRI use, serum albumin, neutrophil-to-lymphocyte ratio, duration of surgery, intraoperative opioids, and fluid balance. Following selection, we assessed multicollinearity to ensure model stability. **Figure 1E** displays the Spearman correlation heat map, which indicates weak correlations among the selected features. This was further quantified in **Table 3**, where the Variance Inflation Factor analysis showed that all seven variables had VIF values well below the threshold of 5. For instance, chronic SSRI use had a VIF of 1.70 and the duration of surgery had a VIF of 1.62, confirming their independence and suitability for multivariate modeling.

**Table 3:** Collinearity analysis of candidate and selected variables.

Variable	VIF (All candidate variables)	VIF (Selected features)	Inclusion in final model
Age	1.4528394726101		
Gender	1.1509384756201		
BMI	1.3247502938471		
ASA classification	2.1503928475629		
Smoking history	1.5203948572610		
Preoperative opioid use	1.6829301928374		
History of depression	1.2058374629103	1.0982374610293	Yes
Chronic SSRI use	1.4829102938472	1.7046186409865	Yes
Sleep quality score (PSQI)	1.8472019384722		
Cognitive score (MMSE)	1.5629304857261		
History of motion sickness	1.2503928475620		
Intraoperative BIS value	1.1820193847265		
Diabetes mellitus	1.5820193847265		
Hypertension or CVD	1.7203948572109		
COPD	1.2938475620193		
History of neurological disease	1.1503928475629		
Serum albumin	2.8472019384751	1.3540293847102	Yes
NLR	3.4203948572619	1.4209384756291	Yes
CRP	3.1029384756201		
Hemoglobin	2.6503928475619		
Preoperative potassium	1.1829304857261		
eGFR	1.4503928475629		
Fasting blood glucose	1.6209384756201		
Duration of surgery	12.847201938472	1.6203948572610	Yes
Duration of anesthesia	13.203948572619		
Spine surgery	1.3503928475620		
Intraoperative opioids (MME)	1.5203948572610	1.2847502938475	Yes
Anesthesia maintenance type	1.2209384756201		
Duration of hypotension	1.4503928475629		
Vasopressor use	1.5809384756201		
NMBA reversal agent	1.1203948572619		
Fluid balance	1.5629304857261	1.2403948572619	Yes
Estimated blood loss	2.1384756201938		
Postoperative PCIA use	1.3820193847261		
Pain score at POD1 (VAS)	1.4820193847261		
Time to first mobilization	1.7503928475620		
Postoperative potassium	1.2203948572619		
Perioperative transfusion	1.9203948572619		

### Variable importance consistency across machine learning models

**Figure 2:**
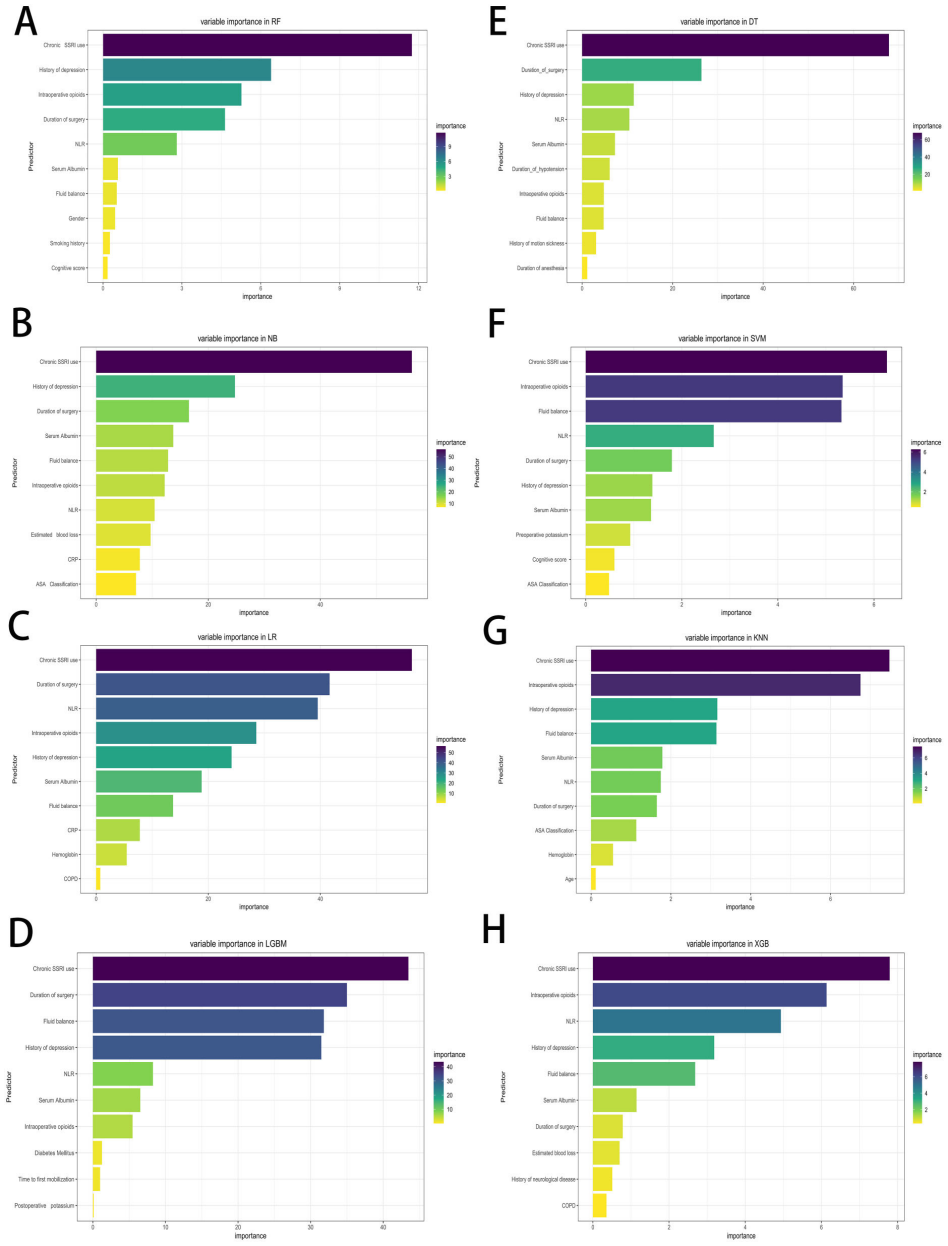
Feature importance ranking across eight machine learning algorithms. Bar charts display the relative contribution of predictors for: **(A)** Random Forest; **(B)** Naïve Bayes; **(C)** Logistic Regression; **(D)** LGBM; **(E)** Decision Tree; **(F)** SVM; **(G)** KNN; **(H)** XGBoost. Color intensity corresponds to the importance score.

We subsequently evaluated the contribution of these seven features across eight different machine learning algorithms. These algorithms included Random Forest, XGBoost, LightGBM, Logistic Regression, Naive Bayes, Decision Tree, Support Vector Machine, and K-Nearest Neighbors. **Figure 2** presents the feature importance rankings for each model. Despite differences in algorithmic principles, chronic SSRI use and a history of depression consistently ranked among the top predictors across most models. This cross-model consistency validates the robustness of the feature selection and underscores the critical influence of brain-gut axis factors on the risk of postoperative ileus.

### Comprehensive model performance in training, testing, and validation cohorts

**Figure 3:**
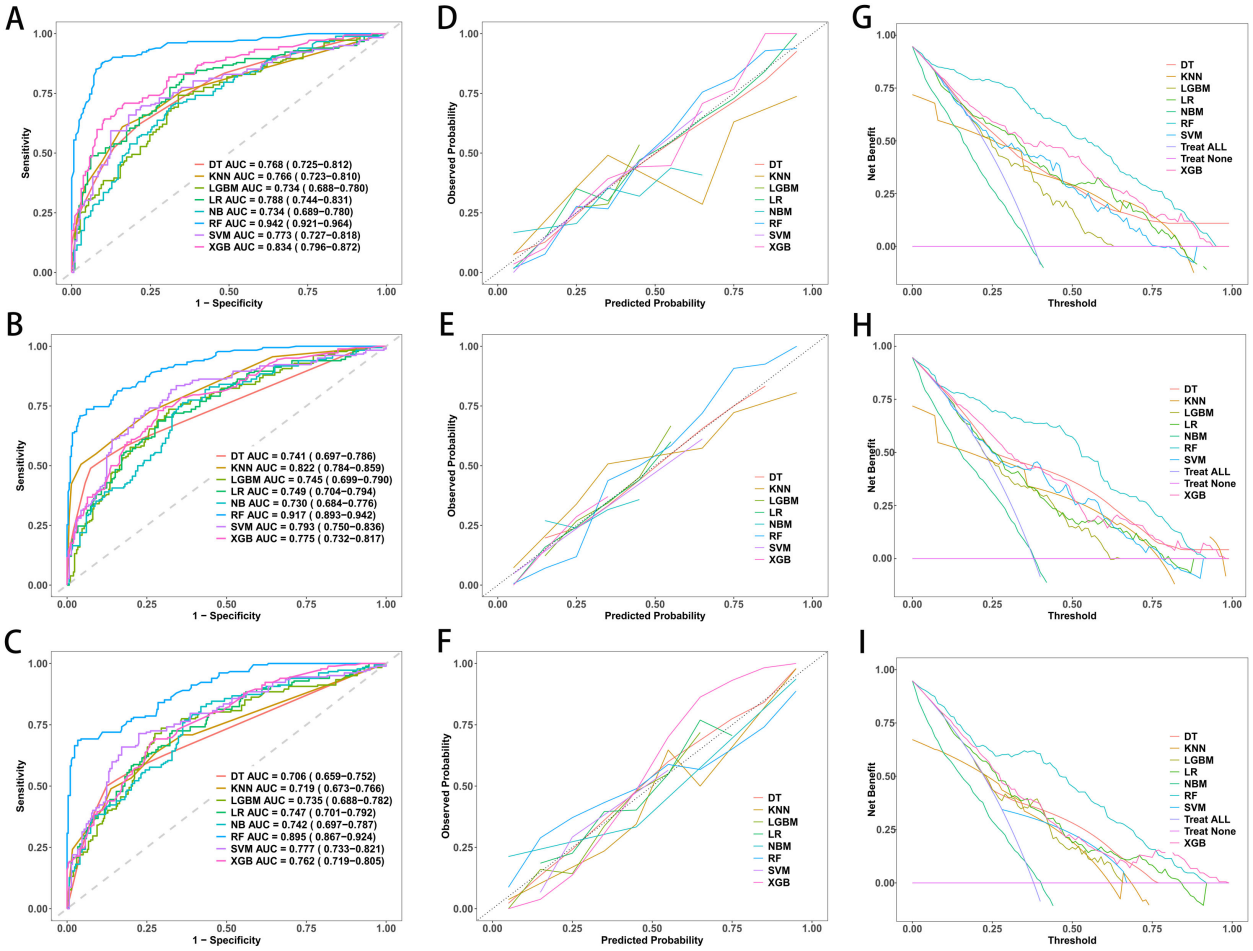
Performance evaluation in training (top row), internal testing (middle row), and external validation (bottom row) cohorts. **(A–C)** ROC curves comparing the discriminatory ability of the eight models. **(D–F)** Calibration plots assessing agreement between predicted probabilities and observed frequencies. **(G–I)** Decision curve analysis (DCA) illustrating the net clinical benefit of the models.

Using the seven identified predictors, we developed and compared the eight machine learning models. **Figure 3** illustrates the performance metrics across all three cohorts. In the ROC analysis shown in **Figures 3A–C**, the Random Forest model achieved the highest discrimination capabilities. Specifically, the Random Forest model yielded an AUC of 0.942 in the training set, 0.917 in the internal testing set, and 0.895 in the external validation set. These values were consistently higher than those of the Logistic Regression model and other comparative algorithms.

We further assessed the calibration and clinical utility of the models. The calibration curves in **Figures 3D-F** demonstrate that the Random Forest model exhibited high agreement between predicted probabilities and observed outcomes, with the curve closely following the ideal diagonal line. Additionally, the Decision Curve Analysis presented in **Figures 3G-I** indicate that the Random Forest model provided the highest net benefit across a broad range of threshold probabilities from 20% to 80%, suggesting superior clinical value compared to default intervention strategies.

Beyond discrimination capabilities, the calibration of all eight models was quantitatively assessed using the Brier score (summarized in **Supplementary Table 1**). The Random Forest model demonstrated the best overall calibration, achieving the lowest Brier scores of 0.056 in the training cohort, 0.071 in the internal testing cohort, and 0.085 in the external validation cohort. Notably, the Random Forest model outperformed the traditional Logistic Regression model (Brier score of 0.128 in external validation) and all other machine learning algorithms, further confirming the superior reliability of its predicted probabilities.

### Statistical comparison and optimization in the training set

**Figure 4:**
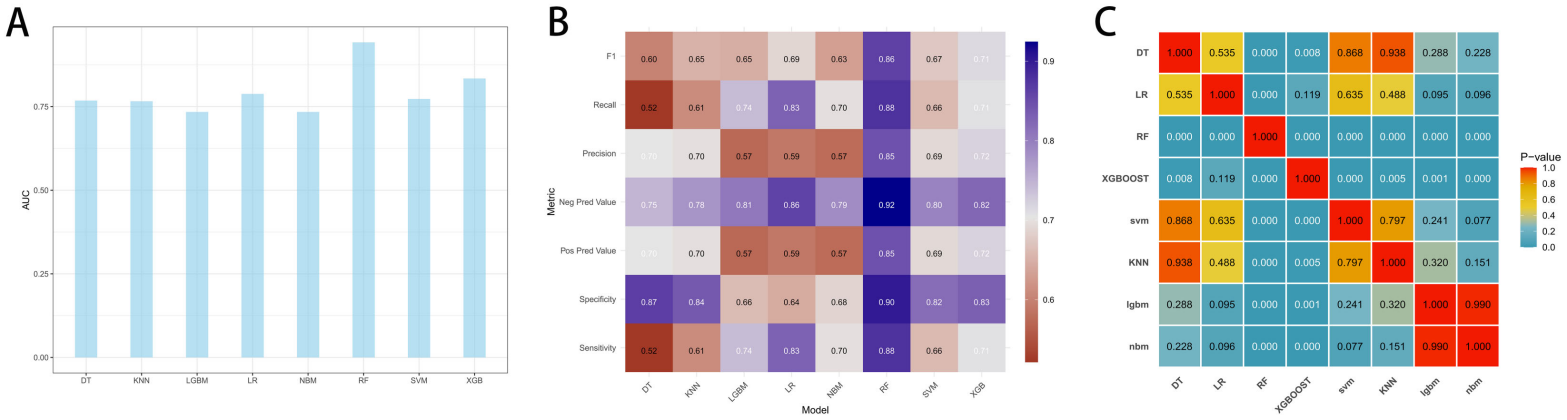
Model performance metrics and statistical comparison in the training cohort. **(A)** Comparison of AUC values among the eight models. **(B)** Heat map of comprehensive performance metrics. **(C)** Heatmap of P-values from pairwise DeLong tests comparing the ROC curves of the 8 machine learning models. The values in the cells represent the raw P-values. Statistical significance was evaluated following Bonferroni correction for multiple comparisons. The RF model’s superiority remained robust and highly significant against all other models.

To statistically confirm the optimal model, we conducted a detailed performance comparison within the training cohort. **Figure 4A** compares the AUC values, highlighting the leading position of the Random Forest model. This is further supported by the heat map in **Figure 4B**, where the Random Forest model displays superior performance across multiple metrics including sensitivity, specificity, and F1-score. We employed the DeLong test to evaluate the significance of these differences. As shown in **Figure 4C**, the Random Forest model yielded significantly higher AUC values compared to Logistic Regression, Naive Bayes, and Decision Tree, with P values less than 0.05. After applying the Bonferroni correction for multiple comparisons, the Random Forest (RF) model demonstrated a statistically significant superiority over all other 7 algorithms (all P < 0.001). Although the difference between Random Forest and XGBoost was smaller, the Random Forest model maintained a statistical advantage, justifying its selection as the final prediction model.

### Robustness validation via cross-validation

**Figure 5:**
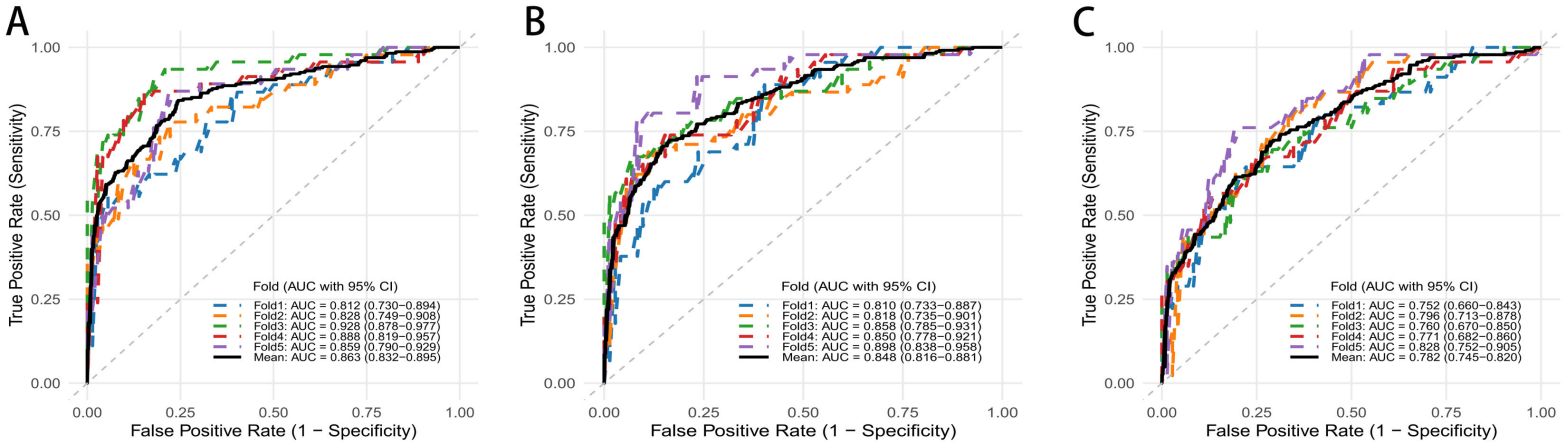
Five-fold cross-validation of the random forest model. ROC curves showing the stability of the model in the: **(A)** Training Cohort; **(B)** Internal Testing Cohort; **(C)** External Validation Cohort. Solid black lines represent the mean ROC, while dashed colored lines represent individual folds.

We performed 5-fold cross-validation to verify the stability of the Random Forest model. **Figure 5** displays the ROC curves for each fold across the training, testing, and validation cohorts. The curves are tightly clustered with minimal variance. In the training cohort shown in **Figure 5A**, the model achieved a mean AUC of 0.863 with a 95% Confidence Interval of 0.832 to 0.895. The internal testing cohort in **Figure 5B** showed a mean AUC of 0.848 with a 95% Confidence Interval of 0.816 to 0.881. Furthermore, the external validation cohort in **Figure 5C** demonstrated a mean AUC of 0.782 with a 95% Confidence Interval of 0.745 to 0.820. These results demonstrate that the model possesses strong generalization capabilities and is not subject to significant overfitting or sampling bias.

### Model interpretability and clinical visualization

**Figure 6:**
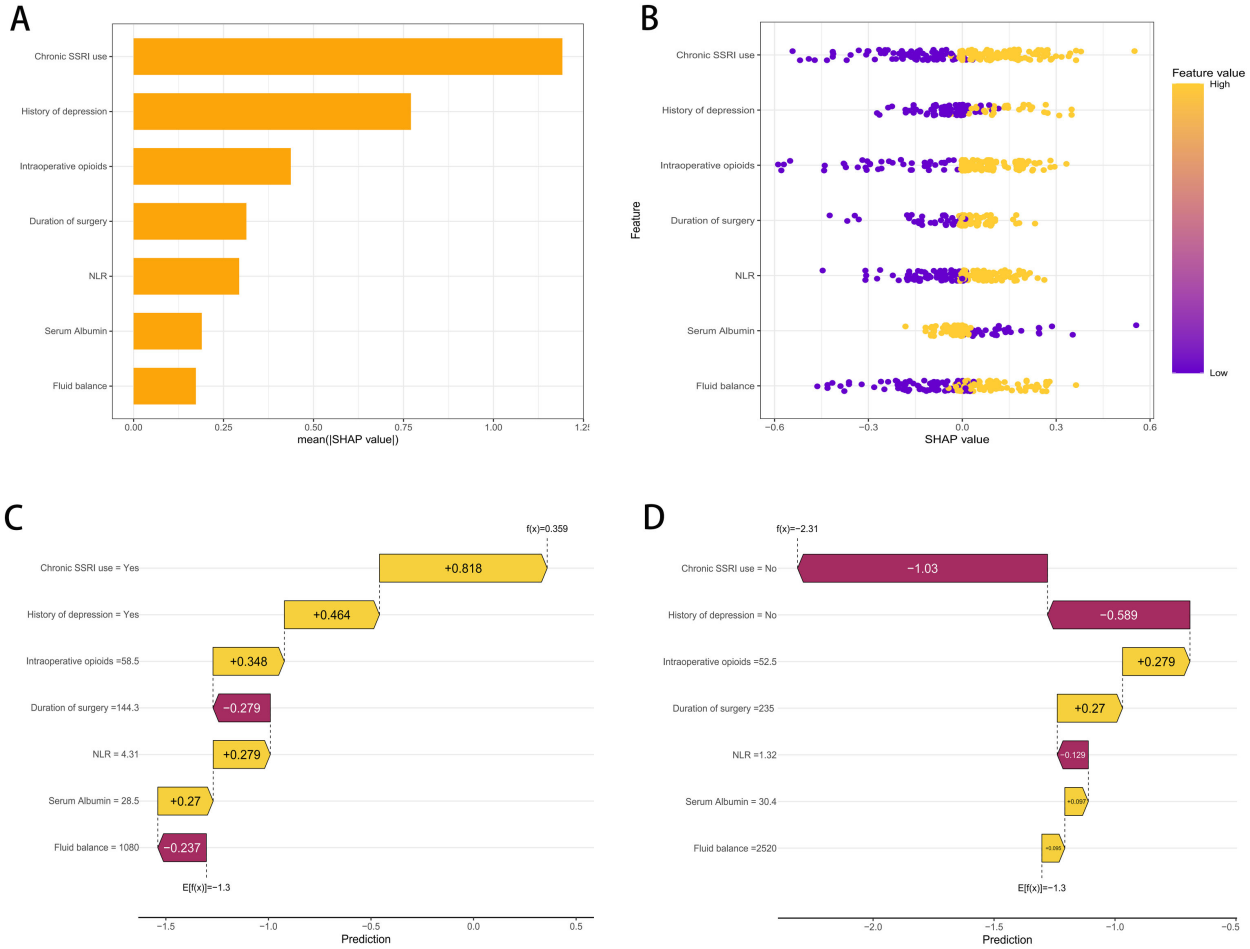
SHAP analysis for random forest model interpretation. **(A)** mean absolute SHAP values ranking global feature importance. **(B)** SHAP summary plot showing the impact of feature values on prediction (color indicates feature value; x-axis indicates impact). **(C)** Waterfall plot illustrating feature contributions for a high-risk patient. **(D)** Waterfall plot illustrating feature contributions for a low-risk patient.

Finally, we utilized SHapley Additive exPlanations to interpret the predictions of the Random Forest model. **Figure 6A** ranks the features by their global mean absolute SHAP values, identifying chronic SSRI use as the most influential predictor. The summary plot in **Figure 6B** elucidates the directional impact of each feature. It reveals that patients with a history of depression, chronic SSRI use, elevated neutrophil-to-lymphocyte ratio, and positive fluid balance typically have higher SHAP values associated with increased risk. Conversely, higher serum albumin levels act as a protective factor. **Figures 6C, D** provide waterfall plots for individual patients, visualizing how specific risk factors linearly contribute to the final probability of postoperative ileus for a high-risk and a low-risk case respectively.

## Discussion

In this multicenter retrospective study, we successfully developed and validated a machine learning model to predict the risk of postoperative ileus in patients undergoing non-abdominal surgery. By employing a rigorous feature selection strategy that combined the LASSO and Boruta algorithms, we identified seven core predictors from a broad array of clinical variables. The Random Forest model demonstrated superior discrimination and calibration capabilities compared to traditional logistic regression and other machine learning algorithms across training, internal testing, and external validation cohorts. A distinguishing feature of our study is the integration of brain-gut axis variables into the predictive framework. Our findings highlight that a history of depression and chronic SSRI use are powerful predictors of postoperative gastrointestinal dysfunction, offering new insights into the systemic regulation of bowel motility.

The identification of chronic SSRI use and a history of depression as top-ranking predictors underscores the critical relevance of the brain-gut axis in the perioperative setting. Serotonin is a pivotal neurotransmitter that regulates both mood in the central nervous system and motility in the enteric nervous system (Henze et al., 2013). Chronic administration of SSRIs can alter the sensitivity of peripheral serotonin receptors and deplete platelet serotonin stores, potentially leading to dysregulated gut contractility (McVey Neufeld et al., 2019). Furthermore, patients with depression often exhibit autonomic dysfunction characterized by reduced vagal tone and sympathetic overactivity (Licht et al., 2009). Since vagal stimulation is essential for promoting intestinal peristalsis, the suppression of parasympathetic activity in depressed patients may biologically predispose them to prolonged postoperative ileus (Huston and Tracey, 2011; Hong et al., 2019). Our model captures these complex neurobiological interactions, which are frequently overlooked in conventional risk assessment tools.

Beyond neuropsychiatric factors, the other five selected variables reflect the inflammatory and metabolic stress of surgery. The neutrophil-to-lymphocyte ratio served as a robust marker of systemic inflammation in our model. Surgical trauma triggers an inflammatory cascade that releases cytokines, which can directly inhibit smooth muscle activity in the gut (Sharma et al., 2017; Ding et al., 2022). Similarly, hypoalbuminemia and positive fluid balance were identified as significant risk factors. Low serum albumin levels often indicate poor nutritional status and can exacerbate visceral edema (Rocha and Fortes, 2015; Abahuje et al., 2020). When combined with excessive fluid administration, this leads to intestinal wall edema, which physically impairs peristalsis and absorption (Bayir and Yildiz, 2015; Zhou et al., 2015; Kaki et al., 2025). The inclusion of intraoperative opioid dosage and duration of surgery further confirms established knowledge that pharmacological inhibition of mu-opioid receptors and prolonged anesthesia are fundamental drivers of gastrointestinal stasis (Luchetti et al., 2020; Hoc et al., 2021; Aoyama et al., 2022).

From a methodological perspective, the superiority of the Random Forest model over Logistic Regression can be attributed to its ability to handle non-linear relationships and high-dimensional interactions (Leha et al., 2019). Biological systems are inherently complex, and the relationship between a risk factor and an outcome is rarely purely linear. For instance, the impact of fluid balance on bowel recovery may vary depending on the patient’s albumin level or inflammatory status. Tree-based ensemble methods like Random Forest can naturally model these conditional dependencies. Moreover, our dual-algorithm feature selection approach ensured that the final model was both parsimonious and robust. By taking the intersection of LASSO, which minimizes overfitting, and Boruta, which preserves all relevant features, we effectively eliminated collinearity while retaining the most informative predictors.

The clinical utility of our findings is enhanced by the implementation of SHAP analysis, which mitigates the “black box” nature of machine learning. The SHAP waterfall plots allow clinicians to visualize the specific contribution of each risk factor for an individual patient (Zhang et al., 2023). This transparency is vital for clinical decision-making. For a patient identified as high-risk due to chronic SSRI use and elevated inflammation, the surgical team might adopt preemptive strategies. These could include the implementation of multimodal opioid-sparing analgesia, more restrictive fluid management, or the early administration of peripheral mu-opioid receptor antagonists. Thus, our model serves not merely as a prediction tool but as a guide for personalized perioperative management. To facilitate the immediate clinical translation of our findings, we have developed a user-friendly, web-based calculator (freely accessible at https://my-portfolio.shinyapps.io/Postoperative_Ileus/). In practical clinical settings, this model could be seamlessly integrated into existing Electronic Health Record (EHR) systems via application programming interfaces. During the perioperative workflow, the EHR system could automatically extract the seven required predictors to generate a real-time POI risk score before the patient is transferred to the ward. This automated risk stratification would not increase the manual workload of clinicians but would serve as a prompt alert system. For patients flagged as high-risk by the model, the surgical team could seamlessly trigger proactive, standardized care pathways, such as tailored fluid management, optimized analgesia protocols, or early mobilization strategies, thereby effectively transforming predictive data into actionable clinical decisions.

While our model highlights the critical role of the brain-gut axis in POI development, translating these findings into clinical practice requires specific, testable interventions. As chronic SSRI use emerged as the top predictor of POI, determining its optimal perioperative management is paramount. Current psychiatric guidelines strongly advise against the abrupt discontinuation or empirical dose-adjustment of SSRIs prior to surgery due to the high risk of withdrawal syndrome and acute psychiatric decompensation, which could further escalate surgical stress. Therefore, we hypothesize that SSRIs should be continued perioperatively, but chronic users must be pre-emptively stratified into a ‘highly vulnerable’ category for POI. For these patients, enhanced monitoring and targeted prophylactic interventions, such as the early administration of prokinetics, targeted sham feeding, and aggressive early enteral nutrition within strict Enhanced Recovery After Surgery pathways, should be prioritized.

Furthermore, the mechanistic link between SSRI use, depression, and POI is heavily mediated by the autonomic nervous system, specifically impaired parasympathetic output. In this context, preoperative assessment of vagal tone could serve as a vital, modifiable biomarker. We propose that evaluating vagal tone non-invasively via Heart Rate Variability (HRV) during the preoperative anesthesia assessment can identify patients with significant autonomic dysfunction. We hypothesize that patients with low preoperative HRV and concurrent SSRI use are at the highest risk for severe POI.

To validate these clinical strategies, specific prospective trials are urgently needed. First, a randomized controlled trial (RCT) should be designed to evaluate whether preemptive, targeted gastrointestinal prophylaxis can significantly reduce the incidence of POI in patients on chronic SSRIs. Second, prospective studies should investigate the therapeutic potential of neuromodulation, such as evaluating whether preoperative HRV-guided transcutaneous auricular vagus nerve stimulation (taVNS) can restore autonomic balance and mitigate the risk of POI in this specific high-risk cohort.

Despite these promising results, this study has several limitations that must be acknowledged. First, the retrospective design introduces potential selection bias and limits our ability to infer causality between brain-gut axis variables and postoperative ileus. While the predictive value of depression and SSRI use in our model is compelling, these findings must be interpreted with caution due to the possibility of residual confounding. Specifically, these variables may be subject to indication bias or confounded by unmeasured factors such as the severity of the underlying psychiatric comorbidities, variations in patients’ subjective pain perception, or subtle differences in perioperative management. Although we adjusted for multiple confounders, unmeasured variables such as intraoperative catecholamine levels or specific surgical techniques might still influence the results. Second, while we validated the model in an independent external center, both hospitals are tertiary care institutions. This may limit the generalizability of our findings to smaller community hospitals with different perioperative protocols. Third, regarding the primary outcome, our definition and diagnostic approach have inherent limitations. We utilized a >4 days threshold to capture prolonged and clinically impactful POI. While this approach effectively targets patients requiring actual medical intervention and prevents over-alerting clinicians for transient physiological bowel delays, we acknowledge that it may miss mild or moderate cases that resolve by postoperative day 3. Future studies could explore multi-class prediction models to differentiate between transient and prolonged ileus. Furthermore, the diagnosis of POI in our cohort relied on a composite of clinical symptoms and chart review, which may be less precise than prospective functional studies using manometry or radiopaque markers. Despite our use of a standardized data extraction protocol across centers, inherent variability in clinical documentation practices and the subjective assessment of symptoms may still introduce a degree of misclassification bias. Despite our use of a standardized data extraction protocol across centers, inherent variability in clinical documentation practices and the subjective assessment of symptoms may still introduce a degree of misclassification bias. Fourth, our approach to handling missing data relied on mean and mode imputation. While this preserved our sample size given the low rate of missingness, single imputation methods fail to account for the uncertainty of missing data and can artificially reduce variable variance. Future studies would benefit from employing more robust techniques, such as Multiple Imputation by Chained Equations (MICE) or conducting sensitivity analyses, to further validate the stability of the predictors.

In conclusion, we constructed a robust Random Forest model that accurately predicts postoperative ileus in non-abdominal surgery patients. By demonstrating the significant predictive value of depression history and chronic SSRI use, our study reinforces the importance of the brain-gut axis in surgical recovery. This model provides a practical and interpretable tool for clinicians to identify high-risk patients and implement targeted interventions, ultimately potentially reducing the incidence of this burdensome complication. Future prospective studies are warranted to validate these findings and evaluate whether psychological prehabilitation can improve postoperative gastrointestinal outcomes.

## Data Availability

The raw data supporting the conclusions of this article will be made available by the authors, without undue reservation.
