# Supplementary material for: Beyond the abdomen: an interpretable machine learning model for predicting postoperative ileus in non-abdominal surgery
Source: Front Physiol. 2026 Jun 12;17:1814394. doi: 10.3389/fphys.2026.1814394 (PMC13303618; doi:10.3389/fphys.2026.1814394)
Supplement: Supplementary file 1 [file Table1.docx]

**Supplementary Table 1.** Brier Scores of the Eight Evaluated Machine Learning Models across Three Cohorts

| **Model** | **Training Cohort** | **Testing Cohort** | **Validation Cohort** |
| --- | --- | --- | --- |
| **Random Forest (RF)** | 0.056 | 0.071 | 0.085 |
| **XGBoost (XGB)** | 0.084 | 0.098 | 0.112 |
| **Logistic Regression (LR)** | 0.102 | 0.115 | 0.128 |
| **Support Vector Machine (SVM)** | 0.108 | 0.121 | 0.134 |
| **Decision Tree (DT)** | 0.112 | 0.125 | 0.138 |
| **K-Nearest Neighbors (KNN)** | 0.115 | 0.128 | 0.141 |
| **Naive Bayes (NB)** | 0.128 | 0.139 | 0.152 |
| **LightGBM (LGBM)** | 0.129 | 0.140 | 0.154 |
