# Supplementary material for: Beyond the abdomen: an interpretable machine learning model for predicting postoperative ileus in non-abdominal surgery
Source: Front Physiol. 2026 Jun 12;17:1814394. doi: 10.3389/fphys.2026.1814394 (PMC13303618; doi:10.3389/fphys.2026.1814394)
Supplement: Supplementary file 2 [file Table2.docx]

**Supplementary Table 2. Missing data rates for candidate variables across the training, testing, and validation cohorts**

| **Variable** | **Training Cohort (N = 1050)** | **Testing Cohort (N = 450)** | **Validation Cohort (N = 500)** |
| --- | --- | --- | --- |
| Age | 0 (0.0%) | 0 (0.0%) | 0 (0.0%) |
| Gender | 0 (0.0%) | 0 (0.0%) | 0 (0.0%) |
| BMI | 12 (1.1%) | 6 (1.3%) | 8 (1.6%) |
| ASA Classification | 0 (0.0%) | 0 (0.0%) | 0 (0.0%) |
| Smoking history | 0 (0.0%) | 0 (0.0%) | 0 (0.0%) |
| Preoperative opioid use | 5 (0.5%) | 2 (0.4%) | 3 (0.6%) |
| History of depression | 0 (0.0%) | 0 (0.0%) | 0 (0.0%) |
| Chronic SSRI use | 0 (0.0%) | 0 (0.0%) | 0 (0.0%) |
| Sleep quality score (PSQI) | 21 (2.0%) | 10 (2.2%) | 14 (2.8%) |
| Cognitive score (MMSE) | 18 (1.7%) | 8 (1.8%) | 11 (2.2%) |
| History of motion sickness | 9 (0.9%) | 4 (0.9%) | 5 (1.0%) |
| Diabetes Mellitus | 0 (0.0%) | 0 (0.0%) | 0 (0.0%) |
| Hypertension or CVD | 0 (0.0%) | 0 (0.0%) | 0 (0.0%) |
| COPD | 0 (0.0%) | 0 (0.0%) | 0 (0.0%) |
| History of neurological disease | 0 (0.0%) | 0 (0.0%) | 0 (0.0%) |
| Preoperative potassium | 15 (1.4%) | 7 (1.6%) | 9 (1.8%) |
| Fasting blood glucose | 19 (1.8%) | 9 (2.0%) | 12 (2.4%) |
| Serum Albumin | 31 (3.0%) | 14 (3.1%) | 17 (3.4%) |
| Hemoglobin | 8 (0.8%) | 5 (1.1%) | 6 (1.2%) |
| NLR | 24 (2.3%) | 11 (2.4%) | 14 (2.8%) |
| CRP | 42 (4.0%) | 17 (3.8%) | 21 (4.2%) |
| eGFR | 11 (1.0%) | 6 (1.3%) | 7 (1.4%) |
| Spine surgery | 0 (0.0%) | 0 (0.0%) | 0 (0.0%) |
| Duration of surgery | 0 (0.0%) | 0 (0.0%) | 0 (0.0%) |
| Duration of anesthesia | 0 (0.0%) | 0 (0.0%) | 0 (0.0%) |
| Intraoperative BIS value | 26 (2.5%) | 12 (2.7%) | 15 (3.0%) |
| Intraoperative Opioids (MME) | 10 (1.0%) | 5 (1.1%) | 7 (1.4%) |
| Anesthesia maintenance type | 0 (0.0%) | 0 (0.0%) | 0 (0.0%) |
| Duration of hypotension | 16 (1.5%) | 8 (1.8%) | 9 (1.8%) |
| Vasopressor use | 0 (0.0%) | 0 (0.0%) | 0 (0.0%) |
| NMBA reversal agent | 0 (0.0%) | 0 (0.0%) | 0 (0.0%) |
| Estimated blood loss | 14 (1.3%) | 7 (1.6%) | 10 (2.0%) |
| Fluid balance | 18 (1.7%) | 9 (2.0%) | 11 (2.2%) |
| Perioperative transfusion | 0 (0.0%) | 0 (0.0%) | 0 (0.0%) |
| Postoperative PCIA use | 0 (0.0%) | 0 (0.0%) | 0 (0.0%) |
| Pain score at POD1 (VAS) | 28 (2.7%) | 13 (2.9%) | 16 (3.2%) |
| Time to first mobilization | 34 (3.2%) | 16 (3.6%) | 19 (3.8%) |
| Postoperative potassium | 38 (3.6%) | 15 (3.3%) | 22 (4.4%) |

*Note: Data are presented as Missing Count (Missing Percentage). Missingness was evaluated separately across the three independent cohorts. Imputation parameters (mean/mode) were strictly calculated from the Training cohort to prevent data leakage. Variables with 0.0% indicate completely recorded data. All candidate variables had missing rates well below the 5% threshold.*
